# Verbesina encelioides: cytotoxicity, cell cycle arrest, and oxidative DNA damage in human liver cancer (HepG2) cell line

**DOI:** 10.1186/s12906-016-1106-0

**Published:** 2016-05-10

**Authors:** Mai M. Al-Oqail, Maqsood A. Siddiqui, Ebtesam S. Al-Sheddi, Quaiser Saquib, Javed Musarrat, Abdulaziz A. Al-Khedhairy, Nida N. Farshori

**Affiliations:** Department of Pharmacognosy, College of Pharmacy, King Saud University, Riyadh, 11451 Kingdom of Saudi Arabia; Zoology Department, College of Science, King Saud University, P.O. Box-2455, Riyadh, 11451 Kingdom of Saudi Arabia; Al-Jeraisy Chair for DNA Research, Zoology Department, College of Science, King Saud University, Riyadh, 11451 Kingdom of Saudi Arabia

**Keywords:** *Verbesina encelioides*, Cytotoxicity, Oxidative stress, ROS generation, MMP, DNA damage

## Abstract

**Background:**

Cancer is a major health problem and exploiting natural products have been one of the most successful methods to combat this disease. *Verbesina encelioides* is a notorious weed with various pharmacological properties.

The aim of the present investigation was to screen the anticancer potential of *V. encelioides* extract against human lung cancer (A-549), breast cancer (MCF-7), and liver cancer (HepG2) cell lines.

**Methods:**

A-549, MCF-7, and HepG2 cells were exposed to various concentrations of (10–1000 μg/ml) of *V. encelioides* for 24 h. Further, cytotoxic concentrations (250, 500, and 1000 μg/ml) of *V. encelioides* induced oxidative stress (GSH and LPO), reactive oxygen species (ROS) generation, mitochondrial membrane potential (MMP), cell cycle arrest, and DNA damage in HepG2 cells were studied.

**Results:**

The exposure of cells to 10–1000 μg/ml of extract for 24 h, revealed the concentrations 250–1000 μg/ml was cytotoxic against MCF-7 and HepG2 cells, but not against A-549 cells. Moreover, the extract showed higher decrease in the cell viability against HepG2 cells than MCF-7 cells. Therefore, HepG2 cells were selected for further studies *viz.* oxidative stress (GSH and LPO), reactive oxygen species (ROS) generation, mitochondrial membrane potential (MMP), cell cycle arrest, and DNA damage. The results revealed differential anticancer activity of *V. encelioides* against A-549, MCF-7 and HepG2 cells. A significant induction of oxidative stress, ROS generation, and MMP levels was observed in HepG2 cells. The cell cycle analysis and comet assay showed that *V. encelioides* significantly induced G2/M arrests and DNA damage.

**Conclusion:**

These results indicate that *V. encelioides* possess substantial cytotoxic potential and may warrant further investigation to develop potential anticancer agent.

**Electronic supplementary material:**

The online version of this article (doi:10.1186/s12906-016-1106-0) contains supplementary material, which is available to authorized users.

## Background

Cancer is one of the main causes of human death in developed and developing countries [1–3]. 15 million new cases of cancer are expected, 70 % of which will be in developing countries by 2020, where governments are less prepared to address the growing cancer burden [4]. Lung cancer, breast cancer, and liver cancer were the most common sites of cancer diagnosed in 2012 among men and women [5]. The growing trend indicates deficiency in the present cancer therapies and the average survival rates are less [6]. An accurate and effective treatment of the cancer is very much required for diagnosis of the specific type of cancer diseases. Every cancer type requires a specific course of therapy that includes one or more modalities such as surgery, and/or radiotherapy, and/or chemotherapy [7, 8]. Therefore, there is an urgent need to explore anti-cancer drugs with higher efficacy with less side effects and an affordable cost [9]. Chemotherapy is the most effective method of cancer treatment that uses chemical substances, especially one or more chemotherapeutic agents [10]. Alternative and complementary medicines play an emerging role in the cancer prevention. Plants are the best alternative, as they provide an infinite puddle of effective agents. Since many years, traditional remedies use phytochemicals to treat various diseases because of their inherent potential to cure diseases [11–16]. Among the alternative traditional approaches, various plant products classified as alkaloids, saponins, triterpenes, glycosides, and polyphenols have shown very promising anticancer properties in both in vitro and in vivo [17, 18].

*Verbesina encelioides* (VE)*,* member of Asteraceae (Sunflower) family, is native to the United States, Mexican Plateau, Europe, and Asia including Saudi Arabia [19]. It is a notorious weed and an ornamental plant with various bio efficacies like antibacterial, antifungal, antiviral, hypoglycemic and implantation activities [20]. Traditionally *V. encelioides* finds use for the treatment of sore gums and hemorrhoids [21]. Phytochemical analysis of *V. encelioides* also revealed the presence of important primary metabolites, sesquiterpenes [22], flavonoids [23], galegine [24] and triterpenoids [25]. However, our literature survey revealed no published reports on the anticancer potential of aerial parts of *V. encelioides*. Thus, the present investigation aimed to explore the anticancer efficacy of *V. encelioides* alcoholic extract on human lung cancer (A-549), human breast cancer (MCF-7), and human liver cancer (HepG2) cell lines.

## Methods

### Cell culture

Human lung cancer (A-549), breast cancer (MCF-7), and liver cancer (HepG2) cell lines obtained from American Type Culture Collection (ATCC; Manassas, VA, USA), were grown in Dulbecco’s modified eagle’s medium (DMEM) supplemented with 10 % fetal bovine serum (FBS), 0.2 % sodium bicarbonate, and antibiotic/antimycotic solution (1 ml/100 ml of medium, Invitrogen, Life Technologies, USA). The cells were maintained in 5 % CO_2_ and 95 % atmosphere at 37 °C. Batches of cells showing more than 98 % cell viability were used in the experiments. The cell viability was assessed by trypan blue dye exclusion assay following the protocol of Pant et al. [26].

### Reagents and consumables

All the chemicals, culture mediums, reagents, and kits were procured from Sigma Chemical Company Pvt. Ltd., St. Louis, MO, USA. Culture wares and other plastic consumables used in the study were procured from Nunc, Denmark.

### Preparation of *V. encelioids* extract

The *V. encelioides* plants used in this study were obtained from Harjah, Najran road, Saudi Arabia in October 2013. Dr. Mohammad Atiqur Rahman, taxonomist of Medicinal, Aromatic, and Poisonous Plants Research Center (MAPPRC), College of Pharmacy, King Saud University, Saudi Arabia identified the plants and a specimen (#16048) is submitted in the herbarium of the King Saud University. The sundried plants were ground and extracted with methanol (3 × 10 L) at room temperature. The combined methanol extract was evaporated under reduced pressure to obtain a thick gummy mass. The *V. encelioides* extract was diluted in dimethylsulphoxide (DMSO) for preparation of the various concentrations for cell viability and other assays.

### Experimental design

A-549, MCF-7, and HepG2 cells were exposed to various concentrations of (10–1000 μg/ml) of *V. encelioides* for 24 h. Further, cytotoxic concentrations (250, 500, and 1000 μg/ml) of *V. encelioides* induced oxidative stress (GSH and LPO), reactive oxygen species (ROS) generation, mitochondrial membrane potential (MMP), cell cycle arrest, and DNA damage in HepG2 cells were studied.

### Cytotoxicity assessments by MTT assay

Percentage cell viability was assessed using the 3-(4, 5-dimethylthiazol-2-yl)-2, 5-diphenyl tetrazolium bromide (MTT) assay following the protocol of Siddiqui et al. [27]. Briefly, 10,000 cells were plated in 96 well plates and were allowed to adhere in CO_2_ incubator at 37 °C for 24 h. Then, cells were exposed to different concentrations (10–1000 μg/ml) of *V. encelioides* extract for 24 h. After the exposure, 10 μl of MTT (5 mg/ml of stock) was added in each well and plates were incubated further for 4 h. The supernatant was discarded and 200 μl of DMSO was added in each well and mixed gently. The developed purple color was read at 550 nm. Untreated sets run under identical conditions served as control.

### Cytotoxicity assessment by Neutral red uptake (NRU) assay

NRU assay was carried out following the protocol of Siddiqui et al. [28]. Briefly, 10,000 cells were plated in 96 well plates and were allowed to adhere in CO_2_ incubator at 37 °C for 24 h. Then, cells were exposed to different concentrations (10–1000 μg/ml) of *V. encelioides* for 24 h. After the exposure, the medium was aspirated and cells were washed twice with PBS, and incubated for 3 h in a medium supplemented with neutral red (50 μg/ml). The medium was then washed off rapidly with a solution containing 0.5 % formaldehyde and 1 % calcium chloride. Cells were further incubated for 20 min at 37 °C in a mixture of acetic acid (1 %) and ethanol (50 %) to extract the dye. The plates were read at 550 nm. The values were compared with the control sets.

### Morphological analysis by phase contrast microscope

Morphological alterations in A-549, MCF-7, and HepG2 cells exposed to *V. encelioides* extract were observed under microscope. All the cells were exposed to 10–1000 μg/ml of *V. encelioides* extract for 24 h. The cell images were taken using an inverted phase contrast microscope at 20× magnification.

### Lipid peroxidation (LPO)

Lipid peroxidation was performed using thiobarbituric acid-reactive substances (TBARS) protocol [29]. Briefly, 1 × 10^5^ cells were plated in 6 well plates and were allowed to adhere in CO_2_ incubator at 37 °C for 24 h. Then, cells were exposed to 250–1000 μg/ml of *V. encelioides* for 24 h. After the exposure, HepG2 cells were collected by centrifugation. Then, cells were sonicated in ice cold potassium chloride (1.15 %) and centrifuged at 3000 × g for 10 min. Resulting supernatant (1 ml) was added to 2 ml of thiobarbituric acid (TBA) reagent (15 % TCA, 0.7 % TBA and 0.25NHCl) and was heated at 100 °C for 15 min in a boiling water bath. Samples were then placed in cold ice and were centrifuged at 1000 × g for 10 min. The absorbance of the supernatant was measured at 550 nm.

### Glutathione (GSH) content

Intracellular GSH content was estimated as described [30]. Briefly, 1 × 10^5^ cells were plated in 6 well plates and were allowed to adhere in CO_2_ incubator at 37 °C for 24 h. Then, cells were exposed to 250–1000 μg/ml of *V. encelioides* for 24 h. After the exposure, HepG2 cells were collected by centrifugation. The cellular protein was precipitated by incubating 1 ml sonicated cell suspension with 1 ml TCA (10 %) on ice for 1 h, followed by 10 min centrifugation at 3000 rpm. Then, supernatant was added to 2 ml of 0.4 M Tris buffer (pH 8.9) containing 0.02 M EDTA, followed by an addition of 0.01 M 5,5′-dithionitrobenzoic acid (DTNB) to a final volume of 3 ml. The tubes were incubated at 37 °C for 10 min in a shaking water bath. The absorbance of the yellow color developed was read at 412 nm.

### Reactive oxygen species (ROS) generation

Intracellular ROS generation was detected using the fluorescent probe DCFH-DA dye, following the protocol described by Bakheet et al. [31]. In brief, 2 × 10^4^ cells were plated in 24 well plates and were allowed to adhere in CO_2_ incubator at 37 °C for 24 h. Then, cells were exposed to 250–1000 μg/ml of *V. encelioides* for 24 h. After the exposure, HepG2 cells (500 μl) were incubated in the dark with DCFH-DA (5 μM) for 60 min at 37 °C. Then, cells were immediately washed twice with PBS and centrifuged at 3600 × g for 5 min at room temperature and cell pellets were further suspended in 500 μl PBS. The fluorescence of cells was recorded under 488 nm excitation. Green fluorescence from DCF was measured in the FL1 Log channel through a 525 nm band-pass filter by a Beckman Coulter flow cytometer (Coulter Epics XL/Xl-MCL, USA). Data were presented as the mean fluorescence (MnXI) of 10,000 cells.

### Mitochondrial membrane potential (MMP)

Flow cytometric measurements of MMP were performed following the method described by Bakheet et al. [31]. Briefly, 2 × 10^4^ cells were plated in 24 well plates and were allowed to adhere in CO_2_ incubator at 37 °C for 24 h. Then, cells were exposed to 250–1000 μg/ml of *V. encelioides* for 24 h. After the exposure, control and treated cells were washed twice with PBS. Then the cells were further incubated with 5 μg/ml of mitochondrial specific fluorescent dye Rhodamine-123 for 1 h at 37 °C in dark. Cells were then washed twice with PBS and finally responded in 500 μl of PBS to measure the fluorescence intensity of Rhodamine-123. The intensity was measured using flow cytometry and expressed as the mean fluorescence intensity (MnXI) of 10,000 cells.

### Cell cycle analysis

Cell cycle arrest was determined following the method [32]. In brief, 2 × 10^4^ cells were plated in 24 well plates and were allowed to adhere in CO_2_ incubator at 37 °C for 24 h. HepG2 cells were exposed to different concentrations (250–1000 μg/ml) of *V. encelioides* for 24 h. After the exposure, cells were harvested and centrifuged at 3000 rpm for 5 min. Cell pellets were washed in 500 μl of PBS. Then, cells were fixed with 500 μl of chilled 70 % ethanol, and further incubated at 4 °C for 1 h. After two successive washes with PBS at 3000 rpm for 5 min, cell pellets were suspended in PBS and stained with 50 μg propiodium iodide (PI)/ml containing 0.1 % Triton X-100 and 0.5 mg/ml RNAase A for 1 h at 30 °C in dark. Fluorescence of the PI was measured by flow cytometer using Beckman Coulter flow cytometer (Coulter Epics XL/Xl-MCL, Miami, USA) through a FL-4 filter (585 nm). For the measurement, 10,000 events were acquired. The data were analyzed by Coulter Epics XL/XL-MCL, System II Software, Version 3.0. Cell debris characterized by a low FSC/SSC was excluded from the analysis.

### DNA damage by comet assay

DNA strand breaks in HepG2 cells exposed to different concentrations (250 to 1000 μg/ml) of *V. encelioides* for 24 h were quantified by comet assay following the method described [32]. In brief, cells (5 × 10^4^ cells/well) were exposed to *V. encelioides* in 24 well plates for 24 h at 37 °C. The cells were washed with serum free medium and were harvested by adding 0.065 % trypsin and incubated at 37 °C. The cell suspension was centrifuged at 3000 rpm for 5 min and the pellet was suspended in 100 μl of PBS. The cells were mixed with 100 μl of 1 % LMA and were layered on one-third frosted slides, pre-coated with NMA (1 % in PBS) and kept at 4 °C for 10 min. After gelling, another layer of 90 μl of LMA (0.5 % in PBS) was added. The cells were lysed in a lysing solution overnight. After washing with TBE buffer, the slides were subjected to electrophoresis in cold TBE (Tris-base, 90 mM; boric acid, 90 mM; Na_2_EDTA, 2.5 mM) buffer. Electrophoresis was performed at 1 V/cm for 30 min (16 mA, 32 V) at 4 °C. All preparative steps were conducted in dark to prevent secondary DNA damage. Each slide was stained with 75 μl of 20 μg/ml ethidium bromide solution for 5 min. The slides were analyzed at 40× magnification (excitation wavelength of 515–560 nm and emission wavelength of 590 nm) using a fluorescence microscope (Nikon Eclipse 80i, Japan) coupled with a charge coupled device (CCD) camera. Images from 100 cells (50 from each replicate slide) were randomly selected and subjected to image analysis using software Comet Assay IV (Perceptive Instruments, Suffolk, UK).

### Statistical analysis

Results are expressed as mean ± standard error of three experiments. Statistical analysis was performed using one-way analysis of variance (ANOVA) and Post-hoc Dunnett’s test was applied to compare values between control and treated groups. The values depicting *p* < 0.05 were considered, as statistically significant.

## Results

### Cytotoxicity assessment by MTT assay

**Fig. 1:**
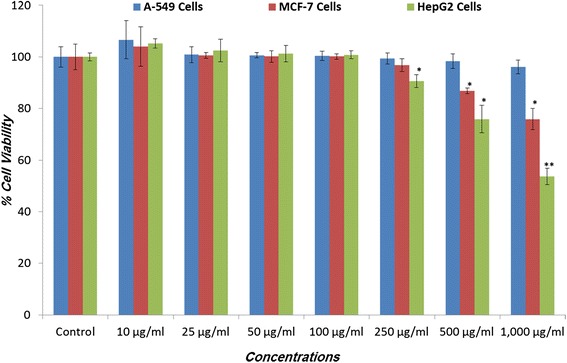
Cytotoxicity assessments by MTT assay in A-549, MCF-7, and HepG2 cells. The cells were exposed to different concentrations of *Verbesina encelioides* extract for 24 h. Values are the mean ± SE of three independent experiments. *p < 0.05 and **p < 0.01 versus Control

Figure 1 shows the results of cytotoxicity assessment of *V. encelioides* extract in A-549, MCF-7, and HepG2 cell lines obtained using MTT assay. A concentration-dependent decrease in cell viability was observed in HepG2 cells following 24 h of exposure to *V. encelioides* extract. The cell viability at concentrations of 250, 500 and 1000 μg/ml of *V. encelioides* extract was measured to be 90, 75 and 53 % in HepG2 cells. Following exposure to 500 and 1000 μg/ml concentrations of *V. encelioides* extract, cell viability was measured to be 87 and 76 % in MCF-7 cells. *V. encelioides* extract was less toxic to MCF-7 cells as compared to HepG2 cells and no cytotoxicity was found even at higher concentrations in A-549 cells (Fig. 1).

### Cytotoxicity assessment byNRU assay

The results obtained using the NRU assay in *V. encelioides* extract treated A-549, MCF-7, and HepG2 cell lines are shown in Additional file 1: Figure S1. A concentration dependent decrease in the cell viability of HepG2 cells was observed following treatment with *V. encelioides* extract for 24 h. The percentage cell viability, after exposure of 250, 500 and 1000 μg/ml of *V. encelioides* was measured to be 91, 72 and 53 % in HepG2 (Additional file 1: Figure S1). In MCF-7 cells, cell viability following treatment with 500 and 1000 μg/ml concentrations of *V. encelioides* extract was recorded as 83 and 72 %. However, A-549 cells treated with *V. encelioides* extract did not cause any decrease in the cell viability (Additional file 1: Figure S1). Like, MTT assay the effect of *V. encelioides* extract was found to be less toxic to MCF-7 cells as compared to the HepG2 cells and non-cytotoxic to A-549 cells (Additional file 1: Figure S1).

### Morphological changes

Additional file 2: Figure S2 shows the morphological changes observed in A-549, MCF-7 and HepG2 cells exposed to different concentrations of *V. encelioides* for 24 h. Morphological changes were observed using phase contrast inverted microscope. The cells exposed to higher doses of *V. encelioides* extract lost their normal morphology and shape; these cells appeared more rounded and less adherent than control. The HepG2 cells indicate the most prominent effects after the exposure of *V. encelioides* as compared to MCF-7 cells. However, A-549 cells exposed to *V. encelioides* extract did not cause any observable change in the normal morphology of the cells (Additional file 2: Figure S2).

### Lipid peroxidation

**Fig. 2:**
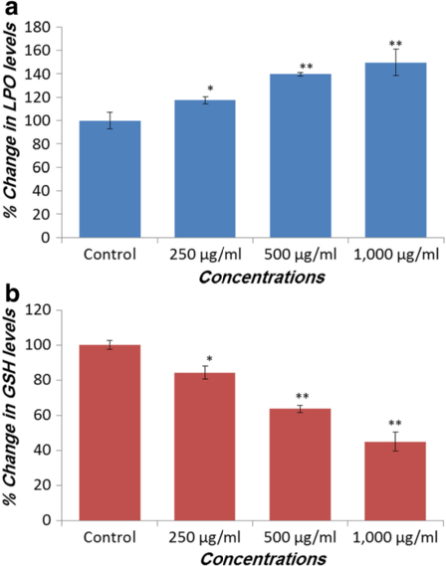
*Verbesina encelioides* extract induced oxidative stress in HepG2 cells exposed for 24 h. **a** Lipid peroxidation; **b** Glutathione depletion. Values are mean ± SE of three independent experiments. *p < 0.05 and **p < 0.01 versus Control

Figure 2a summarizes the effect on lipid peroxidation level induced by *V. encelioides* extract in HepG2 cells. A concentration dependent statistically significant increase in lipid peroxidation was observed. An increase of 17, 39, and 49 % was recorded at 250, 500, and 1000 μg/ml of *V. encelioides* extract, respectively.

### Glutathione depletion

Figure 2b summarizes the decrease in the level of intracellular glutathione in HepG2 cells treated with 250–1000 μg/ml concentrations of *V. encelioides* extract. The results indicate that *V. encelioides* extract decreased the glutathione level in a concentration-dependent manner. The decrease in GSH level was observed as 16, 37, and 56 % at 250, 500, and 1000 μg/ml of *V. encelioides*, respectively as compared to the control (Fig. 2b).

### ROS generation

**Fig. 3:**
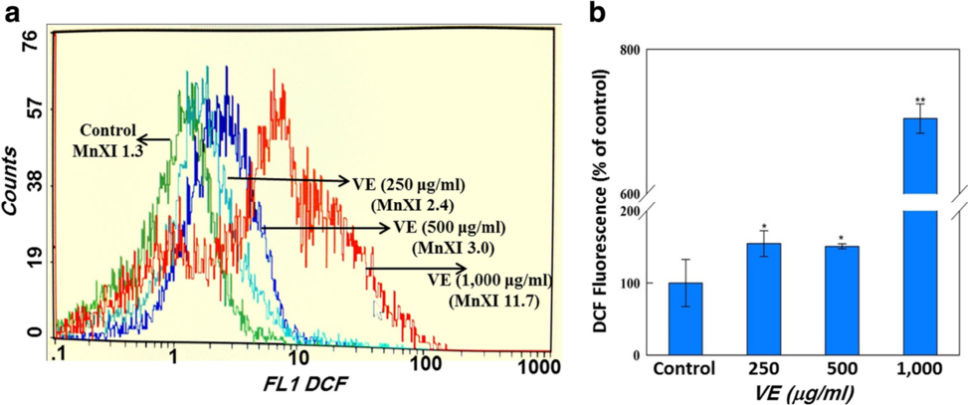
Flow cytometric analysis of intracellular ROS generation in HepG2 cells exposed to *Verbesina encelioides* (VE) extract for 24 h. Panel **a** shows the representative spectra of fluorescent DCF as a function of *Verbesina encelioides* extract concentration. Panel **b** exhibits the comparative analysis of the fluorescence enhancement of DCF with increasing concentrations of *Verbesina encelioides* extract. Each histogram represent the values of mean ± SD of three independent experiments. *p < 0.05, **p < 0.01 versus control

Figure 3 shows the results obtained from ROS generation study. A statistically significant (*p* < 0.01) concentration-dependent ROS generation was observed in HepG2 cells treated with *V. encelioides* extract at 250, 500, and 1000 μg/ml concentrations for 24 h. An increase of 154.63 + 17.55, 150.69 + 3.2, and 704.14 = 20.49 % was observed in ROS generation at 250, 500, and 1000 μg/ml, respectively, as compared to control. However, there was no significant increase observed between the 250 μg/ml and 500 μg/ml concentrations.

### Mitochondrial membrane potential (MMP)

**Fig. 4:**
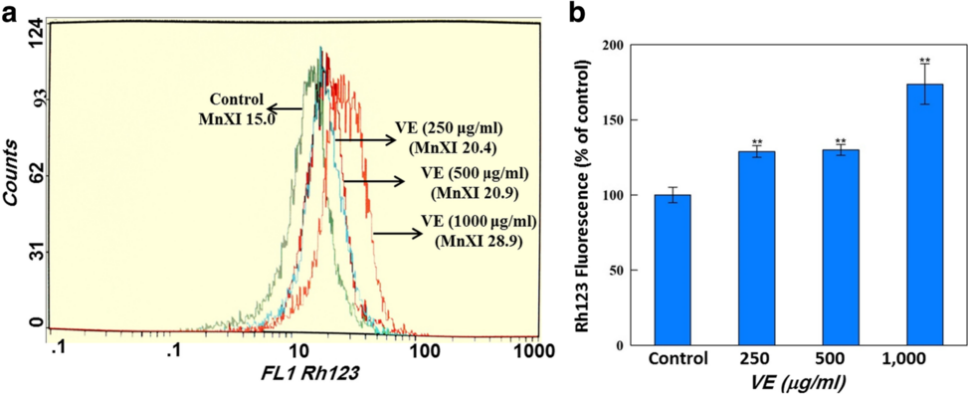
Assessment of mitochondrial membrane potential in HepG2 cells exposed to *Verbesina encelioides* (VE) extract for 24 h. The fluorescence of Rh123was measured using a flow cytometer on log scale with FL1 filter. Panel **a** is a representative FACS image decline in the Rh123 fluorescence as a function of *Verbesina encelioides* extract concentrations. Each histogram in panel **b** represents mean ± S.D. values of Rh123 fluorescence obtained from HepG2 cells exposed to varying concentrations of *Verbesina encelioides* extract. *p < 0.05, ** *p* < 0.01 versus control

Figure 4 depicts the change in the level of MMP. The effect of *V. encelioides* exposure on MMP in HepG2 cells was evaluated. A concentration dependent statistically significant (*p* < 0.01) induction in the level of MMP was also observed in HepG2 cells after the exposure of *V. encelioides* for 24 h. The induction in MMP was observed to be 128.84 ± 3.8, 129.91 ± 3.6, and 173.71 ± 13.43 % at 100, 250, and 500 μg/ml of *V. encelioides*, respectively as compared to untreated control (Fig. 4).

### Cell cycle analysis

**Fig. 5:**
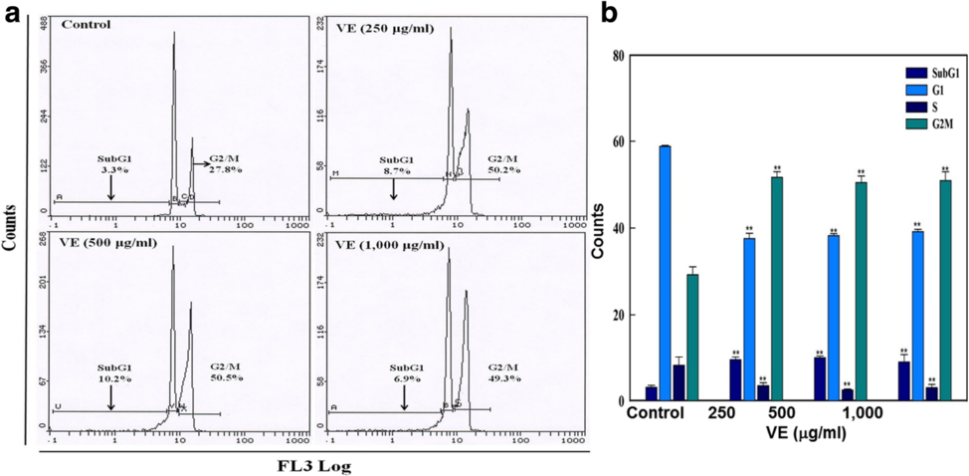
Cell cycle analysis in HepG2 cells exposed to 250–1000 μg/ml concentrations of *V. encelioides* (VE) extract for 24 h. **a** Representative flow cytometric image exhibiting changes in the progression of cell cycle. G2/M in each micrograph represents the percentage of cells in the G2/M phase. **b** Each histogram represents the percentage of cells arrested in different phases of cell cycle. **p < 0.001 vs control

Figure 5 depicts the flow-cytometric analysis of cell cycle progression in HepG2 cells exposed to *V. encelioides* extract for 24 h. Cell cycle analysis of propiodium iodide stained control and *V. encelioides* extract, treated HepG2 cells indicated an increase in apoptotic G2/M peak. At the highest concentration of 1000 μg/ml *V. encelioides* treated cells showed a significant increase of ~50 % in G2/M arrest (Fig. 5).

### DNA damage by comet assay

**Fig. 6:**
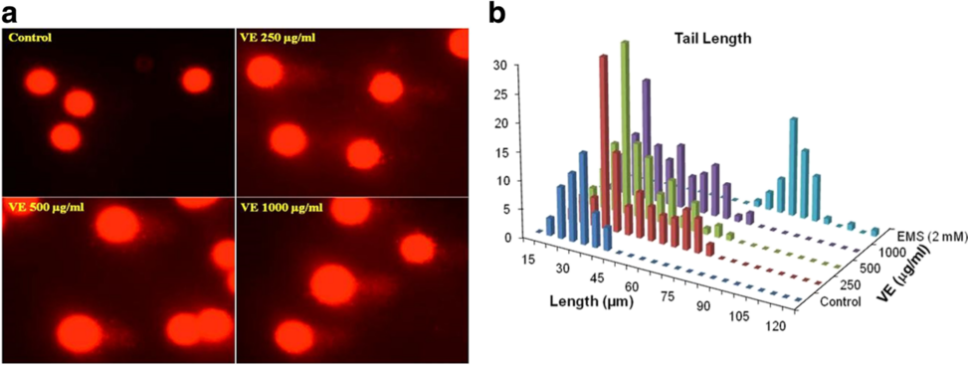
*Verbesina encelioides* extract (VE) induced strand breaks in cellular DNA of HepG2 cells. **a** Representative epi-fluorescence images of DNA damage in comet assay. **b** Percent distribution of DNA damage in HepG2 cells exposed to varying concentrations of *Verbesina encelioides* extract (VE) for 24 h. Olive tail moment (OTM) values were determined following the algorithm (Olive Tail Moment = (Tail Mean – Head Mean) Tail % DNA/100) using Comet Assay IV software. **p < 0.01 versus control

Table 1 summarizes the DNA damage measured by the comet assay parameters i.e., olive tail moment (OTM), tail length, and tail DNA intensity. The HepG2 cells exposed to *V. encelioides* extract for 24 h exhibited significant induction of DNA damage in a concentration dependent manner. The representative images of DNA damage obtained by the comet assay are shown in Fig. 6a and b. The HepG2 cells treated with 250, 500, and 1000 μg/ml of *V. encelioides* exhibited 2.86-, 3.13-, and 3.2-fold higher OTM values, 1.24-, 1.39, and 1.52-fold higher in tail length, and 1.9-, 2.1-, and 2.28-fold higher in tail DNA intensity (Table 1).

**Table 1:** *V. encelioides* (VE) extract induced DNA damage in HepG2 cells after 24 h of exposure, analyzed using different parameters of alkaline comet assay

Groups	Olive tail moment (Arbitrary unit)	Tail length (μm)	Tail intensity (%)
Control	0.38 ± 0.02	34.96 ± 3.87	3.75 ± 0.38
VE extract (μg/ml)			
250	1.09 ± 0.09^*^	43.50 ± 2.45^*^	7.24 ± 0.98^*^
500	1.19 ± 0.07^*^	48.55 ± 3.12^*^	7.84 ± 0.87^*^
1000	1.23 ± 0.14^*^	53.26 ± 4.56^*^	8.55 ± 0.99^*^

Data represent the mean ± S.D. of three independent experiments done in duplicate. *p < 0.05 vs. control. Statistical analysis was performed by one-way analysis of variance (ANOVA) using Dunnett’s multiple comparisons test. The level of statistical significance chosen was *p* < 0.05, unless otherwise stated

## Discussion

Natural products are used for the treatment of various diseases since the beginning of human history. It has been estimated that approximately 80–85 % of the world population rely on traditional medicines for their primarily health care and it is known that a major part of these therapies involves the use of plant extracts or their active components [17, 33, 34]. Most of the chemotherapy medicines to treat the cancer diseases are the molecules identified and isolated from plant materials or their synthetic analogs [35, 36]. Though many researches have been explored for the advancement to protect cancer diseases, still there is a need to develop new drugs to improve the efficacy. The major disadvantages of available synthetic drugs are their association with the side effects [37]. Natural therapy, such as use of the plant products have proved beneficial against the cancer diseases [38, 39]. Thus, there is a constant demand to develop effective new anticancer drugs at affordable cost [36, 40]. Over the last 30 years, natural products have received increasing attention for their potential as new cancer preventive agents [41, 42]. Bearing these facts in mind, we aimed to examine the anticancer potential of *V. encelioides* against three human cancer cell lines, i.e. lung cancer (A-549), breast cancer (MCF-7), and liver cancer (HepG2). Further, oxidative stress (GSH and LPO), reactive oxygen species (ROS) generation, mitochondrial membrane potential (MMP), cell cycle arrest, and DNA damage were also assessed against liver cancer (HepG2) cells. The results of MTT and NRU assay showed that the *V. encelioides* extract exhibited a concentration-dependent cytotoxic response. The MTT and NRU assays are commonly used endpoints for cytotoxicity assessments, since they evaluate different aspects of cellular functions and, therefore, can be useful to examine the potential cytotoxic effects of plant extracts. Estimation of cytotoxicity is generally based on uptake or exclusion of dye and are an indicator of the integrity of the plasma membrane or some intracellular organelles. However, the MTT assay indicates the mitochondrial function based on the enzymatic reduction of a tetrazolium salt by the mitochondrial dehydrogenase of viable cells [43]. NRU is a measure of lysosomal integrity since it reflects the capacity of viable cells to incorporate vital dye into these organelles [44].

The decrease in the cell viability found was specifically towards MCF-7 and HepG2 cells, whereas no effect was observed on A-549 cells. Further, the cytotoxic response was more in HepG2 cells as compared to MCF-7 cells. The differential cytotoxic response of the *V. encelioides* might be due to the specificity of plant towards different cancer cells, as has been reported previously [45, 46]. Our results are well in ordinance with the other findings where these kinds of effects are due to the presence of active components [47]. Our present study demonstrated that *V. encelioides* extract showed promising anti-cancer activity. However, activity was less in MCF-7 cells as compared to HepG2 cells. Thus, HepG2 cells were selected as a model to further investigate the underlying mechanism (s) responsible, for this cytotoxic response. An observable concentration dependent statistically significant increase in lipid peroxidation occurred. An increase of 17, 39, and 49 % was observed at 250, 500, and 1000 μg/ml of *V. encelioides* extract, respectively. Oxidative stress is involved in normal cellular processes of cell signaling [48]. Many studies have shown that exposure to natural products promotes cellular oxidative stress which includes lipid peroxidation and depletion in glutathione levels [49, 50]. Data from this study also showed that *V. encelioides* induced oxidative stress in HepG2 cells in a concentration-dependent manner. An increase in the level of lipid peroxidation and a decrease in the antioxidant enzyme GSH were observed. These findings suggest that oxidative stress may be the primary mechanism of the cell death in HepG2 cells when exposed to *V. encelioides* extract. Previous reports, suggesting the role of oxidative stress in the cell death induced by plant extracts [51, 52] firmly support our results. One of the most common cytotoxic effects in cancer cells is due to the induction of reactive oxygen species (ROS) generation [53]. We found that HepG2 cells exposed to *V. encelioides* extract significantly increased intracellular ROS generation in a concentration-dependent manner. Our results are also in agreement with previous findings, where researchers showed ROS induction due to the treatment of plant extracts [54, 55]. We found an induction in the MMP level in HepG2 cells treated with *V. encelioides* extract for 24 h. Reports suggest that high levels of ROS can lead to cellular damage by resulting in mitochondrial damage, which can then induce cell death [32]. Induction in MMP level, based on cationic fluorescent probe Rh123 indicate the role of oxidative stress and ROS generation in cell death of HepG2 cells, due to the generation of free radicals during the mitochondrial respiration. The results from this study confirmed that HepG2 cells treated with *V. encelioides* extract for 24 h significantly activate the G2/M cell cycle checkpoint as observed by flow-cytometry. Reports reveal that p53 protein is regarded as the guardian of the cell genome and is able to activate cell cycle checkpoints, DNA repair and apoptosis to maintain stability of genome [56]. Flow cytometry analysis of *V. encelioides* extract treated HepG2 cells suggests the activation of DNA repair process as observed by cell cycle arrest in G2/M phase at 250, 500, and 1000 μg/ml concentration of *V. encelioides*. It is also known that the DNA repair mechanisms in the cells are highly conserved, thus the extensive DNA damage may lead to cell-cycle arrest and cell death as observed in this investigation. We have highlighted the DNA damaging potential of *V. encelioides* extract in HepG2 cells by comet assay. The results of Table 1 revealed that the *V. encelioides* extract induced a concentration-dependent significant DNA damage as observed by the induction in the fold change of olive tail moment (OTM), tail length, and tail DNA intensity. This *V. encelioides* extract induced DNA damage can be explained on the basis of the experimental evidence of genotoxic potential in HepG2 cells. The DNA damage may either lead to apoptotic cell death or disrupt the factionalizing of cells [57–59]. The DNA damage induced in HepG2 cells indicates the presence of bioactive components in *V. encelioides* extract that are capable of oxidative DNA damage in cancer cells through their pro-oxidant mechanism.

## Conclusion

The findings from this study, for the first time provide new understandings on the anticancer potential of *Verbesina encelioides. V. encelioides* extract exhibited differential cytotoxic responses in A-549, MCF-7, and HepG2 cell lines. The *V. encelioides* extract exhibit high cytotoxic response in HepG2 cells as compared to MCF-7 cells and proved non-cytotoxic in A-549 cells. Results from this study also entrenched the capacity of *V. encelioides* extract to induce apoptotic cell death in human liver cancer (HepG2) cells through oxidative stress, ROS generation, cell cycle arrest, and DNA damage. Our results suggest that *V. encelioides* extract has promising anticancer potential. Therefore, it can be further used to develop a new anticancer agent to treat the deadly cancer diseases.

## Ethics approval and consent to participate

This information is not relevant.

## Consent for publication

This information is not relevant.

## Availability of data and materials

The datasets supporting the conclusions of this article are included within the article and its additional files.

## Additional files

Additional file 1:Figure S1. Cytotoxicity assessments by NRU assay in A-549, MCF-7, and HepG2 cells. The cells were exposed to different concentrations of Verbesina encelioides extract for 24 h. Values are the mean ± SE of three independent experiments. **p*<0.05 and ***p*<0.01 versus Control. (JPG 394 kb) Additional file 2: Figure S2. Morphological changes in A-549, MCF-7, and HepG2 cells. The cells were exposed to different concentrations of Verbesina encelioides extract for 24 h. Images were taken using an inverted phase contrast microscope at 20× magnification. (JPG 384 kb)

## Abbreviations

*%*: percentage
°C: *degree celsius*
A-549: human lung cancer cell line
CO_2_: carbon dioxide
DCFH-DA: dichloro-dihydro-fluorescein diacetate
DMEM: Dulbecco’s modified eagle’s medium
DMSO: dimethylsulphoxide
DNA: deoxyribonucleic acid
DTNB: (5,5-dithio-bis-(2-nitrobenzoic acid)
EDTA: ethylenediaminetetraacetic acid
FBS: fetal bovine serum
GSH: glutathione
H: hour
HCl: hydrochloric acid
HepG2: human liver cancer cell line
L: liter
*LMA*: *low melting agarose*
LPO: lipid peroxidation
MCF-7: human breast cancer cell line
*mg*: milligram
*ml*: milliliter
MMP: mitochondrial membrane potential
MTT: 3-(4, 5-dimethylthiazol-2-yl)-2, 5-diphenyl tetrazolium bromide
nm: nanometers
NMA: *normal melting agarose*
NRU: neutral red uptake
PBS: phosphate buffer saline
*pH*: potential of hydrogen
PI: propiodium iodide
Rh 123: rhodamine-123
RNAase: ribonuclease
ROS: reactive oxygen species
TBA: thiobarbituric acid
*TBE*: tris/borate/EDTA
TCA: *trichloroacetic acid*
VE: *Verbesina encelioides*
*μg*: microgram
*μl*: microliter
