# Supplementary figures and images for: Verbesina encelioides: cytotoxicity, cell cycle arrest, and oxidative DNA damage in human liver cancer (HepG2) cell line
Source: BMC Complement Altern Med. 2016 May 10;16:126. doi: 10.1186/s12906-016-1106-0 (PMC4862229; doi:10.1186/s12906-016-1106-0)

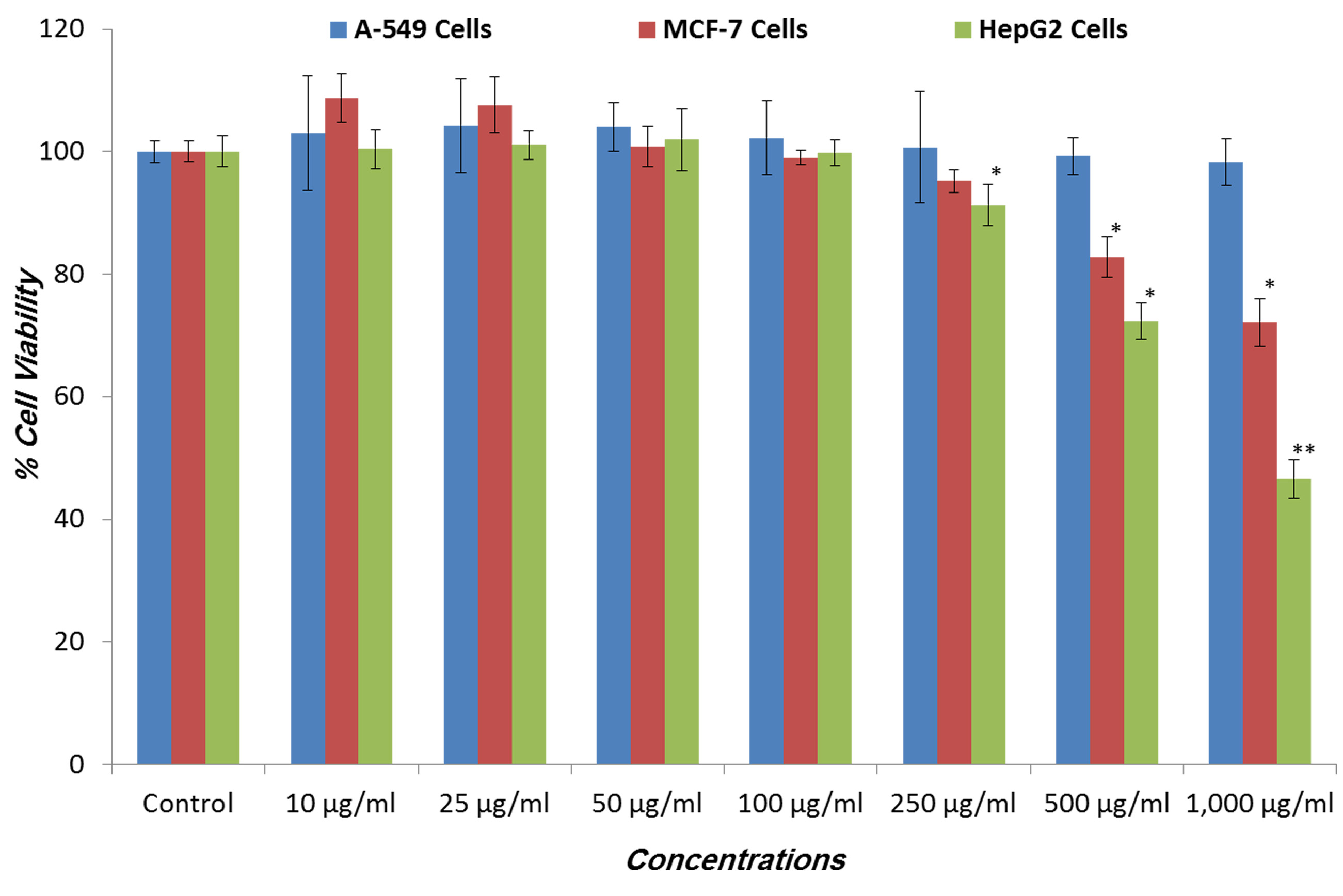

Supplement: Additional file 1:Figure S1. — Cytotoxicity assessments by NRU assay in A-549, MCF-7, and HepG2 cells. The cells were exposed to different concentrations of Verbesina encelioides extract for 24 h. Values are the mean ± SE of three independent experiments. *p<0.05 and **p<0.01 versus Control. (JPG 394 kb) [file 12906_2016_1106_MOESM1_ESM.jpg]

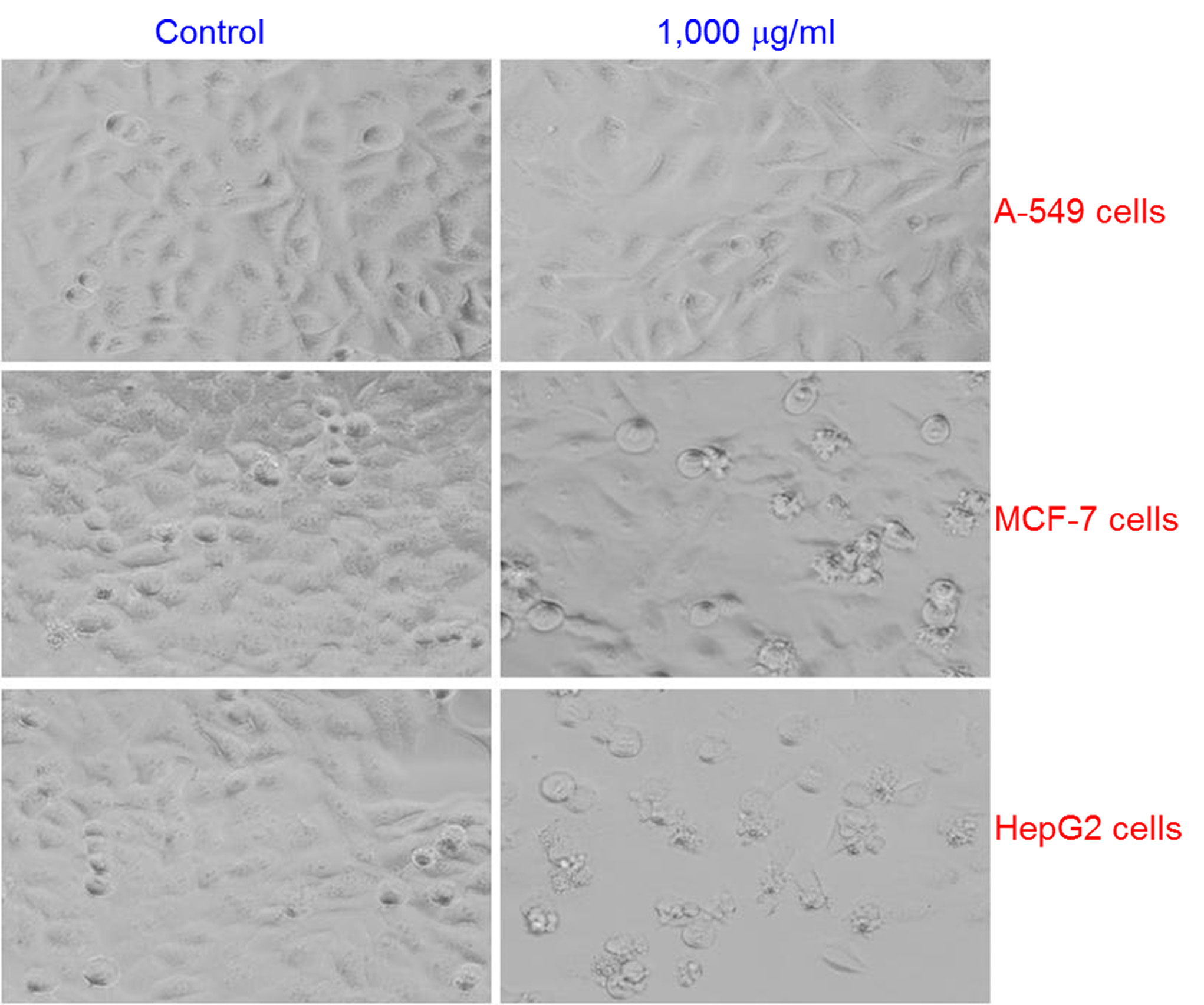

Supplement: Additional file 2: Figure S2. — Morphological changes in A-549, MCF-7, and HepG2 cells. The cells were exposed to different concentrations of Verbesina encelioides extract for 24 h. Images were taken using an inverted phase contrast microscope at 20× magnification. (JPG 384 kb) [file 12906_2016_1106_MOESM2_ESM.jpg]
